# Skeletal Muscle Change During Neoadjuvant Therapy and Its Impact on Prognosis in Patients With Gastrointestinal Cancers: A Systematic Review and Meta-Analysis

**DOI:** 10.3389/fonc.2022.892935

**Published:** 2022-05-27

**Authors:** Xin-Yi Xu, Xiao-Man Jiang, Qin Xu, Hao Xu, Jin-Hua Luo, Cui Yao, Ling-Yu Ding, Shu-Qin Zhu

**Affiliations:** ^1^ Faculty of Health, Queensland University of Technology, Brisbane, QLD, Australia; ^2^ School of Nursing, Nanjing Medical University, Nanjing, China; ^3^ Department of Gastric Surgery, The First Affiliated Hospital of Nanjing Medical University, Nanjing, China; ^4^ Department of Thoracic Surgery, The First Affiliated Hospital of Nanjing Medical University, Nanjing, China; ^5^ Department of Colorectal Surgery, The First Affiliated Hospital of Nanjing Medical University, Nanjing, China

**Keywords:** skeletal muscle mass (SMM), neoadjuvant therapy (NAT), prognosis, gastrointestinal cancers (GI cancers), meta-analysis

## Abstract

**Background:**

Gastrointestinal cancers are the most common malignant tumors worldwide. As the improvement of survival by surgical resection alone for cancers is close to the bottleneck, recent neoadjuvant therapy has been emphasized and applied in the treatment. Despite the advantage on improving the prognosis, some studies have reported neoadjuvant therapy could reduce skeletal muscle and therefore affect postoperative outcomes. However, the conclusions are still controversial.

**Methods:**

PubMed, CINAHL, Embase, and Cochrane Library were searched from inception to September 2, 2021. The inclusion criteria were observational studies, published in English, of individuals aged ≥18 years who underwent neoadjuvant therapy with gastrointestinal cancers and were assessed skeletal muscle mass before and after neoadjuvant therapy, with sufficient data on skeletal muscle change or the association with clinical outcomes. Meta-analysis was conducted by using the STATA 12.0 package when more than two studies reported the same outcome.

**Results:**

A total of 268 articles were identified, and 19 studies (1,954 patients) were included in the review. The fixed effects model showed that the risk of sarcopenia increased 22% after receiving neoadjuvant therapy (HR=1.22, 95% CI 1.14, 1.31, Z=4.286, P<0.001). In the random effects model, neoadjuvant therapy was associated with skeletal muscle loss, with a standardized mean difference of -0.20 (95% CI -0.31, -0.09, *Z*=3.49, P<0.001) and a significant heterogeneity (*I^2 =^* 62.2%, *P*<0.001). Multiple meta regression indicated that population, neoadjuvant therapy type, and measuring tool were the potential sources of heterogeneity. The funnel plot revealed that there was no high publication bias in these studies (Begg’s test, P=0.544) and the sensitivity analysis showed stable results when separately excluding studies. For the postoperative outcomes, the results revealed that muscle loss during neoadjuvant therapy was significantly related to overall survival (HR=2,08, 95% CI =1.47, 2.95, *Z*=4.12, P<0.001, *I^2^ = *0.0%), but not related to disease-free survival and other short-term outcomes.

**Conclusions:**

This systematic review and meta-analysis revealed that skeletal muscle decreased significantly during neoadjuvant therapy in patients with gastrointestinal cancers and skeletal muscle loss was strongly associated with worse overall survival. More high-quality studies are needed to update and valid these conclusions in a more specific or stratified way.

**Systematic Review Registration:**

[https://www.crd.york.ac.uk/PROSPERO/], identifier PROSPERO (CRD42021292118)

## Introduction

Gastrointestinal (GI) cancers, which mainly include esophageal, gastric, and colorectal cancers are commonly diagnosed worldwide and have constituted a heavy disease burden. According to the latest statistics, these three cancers account for 18.7% and 22.6% of the newly cancer cases and deaths, respectively (1). Although surgery is still the main treatment for GI tumors, the improvement of survival by surgical resection alone for locally advanced tumors has been close to the bottleneck (2). Over the past decades, the effect of neoadjuvant therapy (NAT) has been gradually emphasized. NAT includes neoadjuvant chemotherapy (NACT), neoadjuvant chemoradiotherapy (NACRT), targeted therapy and even immunotherapy (2). It was first put forward by Frei in 1982 and was a comprehensive treatment model developing gradually, based on postoperative adjuvant radiotherapy and chemotherapy (3). As recommended by National Comprehensive Cancer Network (NCCN) and American Society of Clinical Oncology (ASCO), surgery combined with NAT has gradually become the standard treatment for patients with local advanced GI cancers (4).

Skeletal muscle could be the largest organ of the human body and plays an important role in maintaining fitness and health status (5). Usually, sarcopenia is defined as the progressive loss of muscle mass and function and mainly involves the elderly; however, researchers have found that volumetric muscle loss (VML), which refers to the rapid-onset and focal loss of skeletal muscle, could exert negative effects on other people as well (6, 7). In cancer patients, except for age-related muscle loss, both tumors and tumor-related treatments are vital reasons for muscle homeostasis imbalance and volumetric muscle loss. Cancer-released exosomes could promote proinflammatory cytokines and factors to elicit activation of cascades and inhibit the function of the muscle metabolism pathways; cancer cells may also activate the ubiquitin-proteasome system (UPS) and the myostatin pathway, as well as increase the expression of phosphatase and tensin homolog (PTEN), therefore leading to protein degradation and muscle atrophy (7). In addition, cancer-related treatments, such as chemotherapy, can trigger off a progressive erosion of the muscle. The potential mechanisms lie in that the molecular pathways of muscle metabolism could be modified by chemotherapeutic drugs directly, therefore inducing sarcopenia (8). Furthermore, chemotherapy or radiotherapy may worsen dysphagia, anorexia, and other cancer-related symptoms, detrimentally affecting nutritional status which could lead to reduced body weight and muscle mass (9).

Numerous studies have shown that low skeletal muscle mass can be a predictor of poor outcomes for cancer patients, which is strongly related to a longer hospital stay, more postoperative complications, reduced survival rates and worse, health-related quality of life (7, 10, 11). Due to the increased consumption of nutrients and metabolic disorders, patients with GI tumors are more likely to suffer from muscle depletion (11), which is strongly associated with worsening of the nutritional state and quality of life in these patients (12). Although NAT has the advantage of reducing tumor size and stage, increasing the R0 resection rate and inhibiting micrometastases (13–15), some studies found skeletal muscle mass could decrease significantly after NAT, which may contribute to preoperative sarcopenia and exacerbate postoperative outcomes (16–18). Currently, various assessment tools exist for the characterization and quantification of muscle loss with easy access in the clinical practice (6), and several strategies, such as ghrelin, oral supplementation, and enteral nutrition or exercise have been found effective to improve muscle mass and attenuation induced by chemotherapy (7, 8). Hence, it is necessary to focus on changes of skeletal muscle mass in patients with GI cancers during NAT, which could provide important evidence for the implementation of early individualized rehabilitation interventions.

Previous meta-analyses have demonstrated that sarcopenia could be a predictor of poor postoperative outcomes in cancer patients (19–21). A recent meta-analysis also reported that NAT could significantly increase the prevalence of sarcopenia in patients with esophageal cancer (EC), and overall survival (OS) and disease-free survival (DFS) were worse in EC patients diagnosed with sarcopenia preoperatively than those who were not (22). However, these studies either failed to consider the impact of NAT itself on skeletal muscle mass or confused the effect of pre-existing sarcopenia with NAT-related sarcopenia on postoperative outcomes. Additionally, whether NAT would reduce skeletal muscle mass in patients with GI cancers and, therefore, affect postoperative outcomes are uncertain. Some studies found no statistically significant reduction in skeletal muscle after NAT (23–25), and muscle loss after NAT may not be a predictor for long-term outcomes in cancer patients (26). Furthermore, findings on short-term outcomes like postoperative complications, and mortality were also contradictory (27–30). Therefore, the purpose of this systematic review and meta-analysis was to evaluate the effect of NAT on skeletal muscle mass in patients with GI cancers and to explore the relationship between muscle change during NAT and clinical outcomes.

## Methods

This systematic review and meta-analysis followed a predefined protocol registered with PROSPERO (CRD42021292118) (31) and adhered to the latest Preferred Reporting Items for Systematic Reviews and Meta-Analyses (PRISMA) guidelines (32).

### Literature Search

A pre-search was conducted in PubMed to identify the subjects, keywords, and synonyms. Then, the PubMed, CINAHL, Embase, and Cochrane Library were searched from inception to September 2, 2021. The search strategies were based on keywords, and the medical subject headings (MeSH) according to PICO framework (**Table 1**) (33). Reference lists of the selected relevant studies were also scanned to further identify additional studies. Search results were imported into Endnote X7 and duplicates were discarded.

**Table 1:** Search strategies according to PICO framework.

Indicator	Description	Search strategies
P (Population)	Patients with esophageal/gastric/colorectal cancer	**MeSH Term:** colorectal neoplasms OR esophageal neoplasms OR stomach neoplasms
		**Keywords:**
		cancer OR tumor OR neoplasm OR tumour OR carcinoma digestive OR gastrointestinal OR gastric OR stomach OR colon OR rectum OR colorec* OR esophag* OR oesophag*
I (Intervention/exposure)	Neoadjuvant therapy including chemotherapy and chemoradiotherapy	**MeSH term:** neoadjuvant therapy
		**Keywords:** “neoadjuvant therapy” OR “neoadjuvant chemotherapy” OR “neoadjuvant chemoradiotherapy”
O (outcome)	Skeletal muscle change	**MeSH Term:** muscle, skeletal
		**Keywords:** sarcopenia OR “muscle wasting” OR “muscle loss” OR “muscle depletion” OR “muscle mass” OR “skeletal muscle” OR “cachexia”

The symbol * means the wildcard symbol that broadens a search by finding words that start with the same letters.

### Eligibility Criteria

We included studies that met these criteria: (1) any original observational studies; (2) included patients who were over 18 years and underwent NAT (including chemotherapy and chemoradiotherapy) with gastric, esophageal, or colorectal cancer; (3) patients were assessed skeletal muscle before and after NAT, and the second assessment must have been completed before any additional treatment like surgery and postoperative chemotherapy; (4) reported sufficient data on skeletal muscle change (providing specific pre-NAT and post- NAT value or change rate) with or without the association with clinical outcomes (e.g., complication, length of hospital stay, mortality, survival rate); (5) only published in English. Studies were excluded if full-text of the studies were unavailable. If studies were based on the same patients, the most recent or completed one was chosen.

### Study Selection and Data Extraction

Firstly, the title and abstract were screened by XX based on the above criteria. The full-text of potential eligible articles were retrieved and evaluated by two researchers (XX and XJ). Papers that did not meet the criteria were excluded, with the reason(s) for exclusion recorded. Any disagreement would be resolved by discussion until consensus was reached or by consulting a third author (SZ). After that, two researchers extracted data by using a standardized data collection form independently. The following data were collected: the first author’s last name, publication year, country, study design, study aim, sample size, sex, age, cancer type and stage, treatment, measuring tool, muscle change outcomes including the degree of muscle loss during NAT, change rate in muscle mass (mean/range), cut-off value and incidence of severe muscle loss, the prevalence of sarcopenia before and after NAT, and findings on the associations between muscle change and clinical outcomes.

### Quality Assessment

The quality of study was independently assessed by two researchers using the National Institutes of Health (NIH) Quality Assessment tool for Observational Cohort and Cross-Sectional Studies. The quality of study was rated as good, fair, or poor (https://www.nhlbi.nih.gov/health-topics/study-quality-assessment-tools).

### Statistical Analysis

Meta-analysis was conducted by using the STATA 12.0 package (StataCorp, College Station, TX, USA) when more than two studies reported the same outcome. To compare the change in the prevalence of sarcopenia before and after NAT, hazard risk (HR) was applied. To compare the change of skeletal muscle during NAT, mean and standard deviation (SD) were applied directly from the publication where possible. For studies that only provided the value of median, range, or interquartile range (IQR), method put forward by Wan et al., was applied to estimate the sample mean and SD (34). Due to the various measurement units of muscle mass, the results were pooled by standardized mean difference (SMD). To analyze the relationship between muscle loss and prognosis, odds ratio (OR) or HR for dichotomous or survival variables were pooled using a fixed or random effects model as appropriate. Heterogeneity of the included studies was assessed by *I^2^* test, and regarded as low, moderate, or high when *I^2^* was around 25%, 50% or 75%, respectively. The random effects model was used when *I^2^* >50%; otherwise, a fixed effects model would be applied (35). When *I^2^* >50%, multiple meta regression was performed to explore whether the heterogeneity could be explained by study design, population, cancer type, NAT type, or measuring tool. The following subgroup analyses was used to illustrate the source of heterogeneity identified by meta regression.

Moreover, sensitivity analysis was conducted by sequentially removing each study, and Begg’s test was used to evaluate publication bias. A two-sided *P* value <0.05 was defined as statistical significance in this study. For those postoperative outcomes that could not be combined, descriptive analysis would be applied.

## Results

### Search Results and Study Characteristic

**Figure 1:**
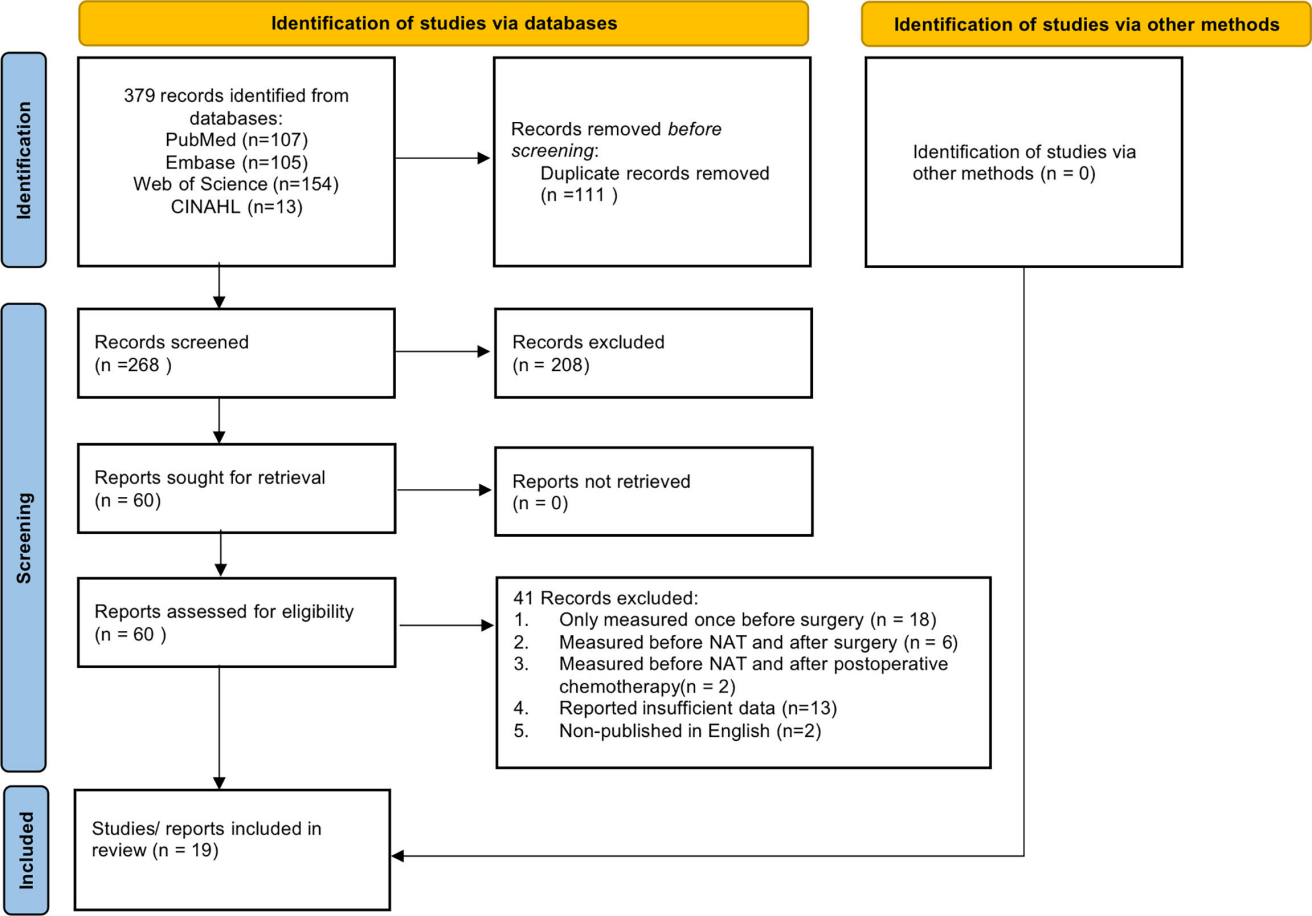
The flow diagram of the study selection process.

A total of 268 articles were found after removal of duplications. Finally, 19 literatures (16–18, 23–27, 29, 30, 36–44) were included in our study with a total sample size of 1,954 participants. The flow diagram of the selection process was presented in **Figure 1**. Among these studies, there were 3 prospective studies (38, 40, 42), while others are retrospective studies. Thirteen studies (18, 24, 26, 29, 30, 37–44) focused on patients with esophageal cancer, one (27) focused on gastro- esophageal cancer, two (25, 36) focused on gastric cancer and three (16, 17, 23) focused on rectal cancer. Furthermore, more than half of the studies (18, 23–26, 29, 36–38, 41) were conducted in the Asian population, with the remaining nine from non-Asian population (16, 17, 27, 30, 39, 40, 42–44). Most studies have limited NAT to neoadjuvant chemotherapy (NAC) or neoadjuvant chemoradiotherapy (NCRT), while 7 studies (16, 25, 29, 30, 41–43) included both therapies. In addition, computed tomography (CT) was the most used tool to measure muscle mass, and only three studies (18, 24, 38) used bioelectrical impedance analysis (BIA). The characteristics of the included studies are shown in **Tables 2**, **3**.

**Table 2:** Main characteristics of included studies (n = 19).

1st Author Year Country	Study design, Aim	Cancer type, stage^1^	Sample size (number of males), age^2^	Treatment^3^	Measuring tool, component^4^	Pre-NAT	Post-NAT	Findings
Yip 2014 UK (39)	Retrospective, To evaluate changes in body composition after NAC and the association with outcomes.	EC	35 (30)	NAC:	CT FFM (kg/m^2^)	18.47 ± 2.24	17.57 ± 2.14	*Muscle change:*
		0-III	63 (34-78)	100%				FFM ↓ significantly after NAC, change rate was -4.6 ± 6.8%. Prevalence of sarcopenia: increased, from 16% to 43%.
								*Association with outcomes:*
								• Skeletal muscle loss (SML) was associated with risk of circumferential resection margin positivity, but not related to survival.
Ida 2014 Japan (38)	Prospective, To determine the influence of NAC on the body composition and to evaluate the association with postoperative complications.	EC	30 (25)	NAC: 100%	BIA SMM (kg)	24.9 ± 0.8	25.2 ± 0.7	*Muscle change:*
		I-IV	65 (53-75)					No significant decrease showed in SMM after NAC. 36.7% patients had SML.
								*Association with outcomes:*
								• Change in skeletal muscle was associated with postoperative complications.
Reisinger 2015 Netherlands (40)	Prospective, To investigate whether the degree of muscle mass lost during NCRT predicts postoperative mortality.	EC	96 (80)	NCRT: 100%	CT	50.9 ± 8.5	48.4 ± 8.5	*Muscle change:*
		I-IV	NS		SMI (cm^2^/m^2^)			SMM ↓ significantly after NCRT. Prevalence of sarcopenia: increased, from 56% to 67%.
								*Association with outcomes:*
								No significant association between muscle loss and mortality was found in the complete cohort. For advanced stage (III-IV), SML may predict postoperative mortality.
Liu 2016 Japan (41)	Retrospective, To determine whether changes in skeletal muscle after NAT predict prognosis.	EC	84 (72)	NAC: 23%	CT SM (PMI) (cm^2^/m^2^)	4.63 (1.77-6.89)^a^	4.54 (1.59-6.89)^a^	*Muscle change:*
		I-III	NS	NCRT: 77%				SMM ↓ significantly after NAT. 64% patients had SML. Cut-off value for severe SML: >-0.28; 50% patients had severe SML.
								*Association with outcomes:*
								• Severe SML during NAT was associated with poor overall survival (OS).
Miyata 2017 Japan (24)	Retrospective, To investigate changes in body composition during NAC and assess whether chemotherapy-related toxicities affect body composition.	EC	94 (76)	NAC: 100%	BIA SMM (kg)	25.0 ± 4.8	24.9 ± 4.8	*Muscle change:*
		I-IV	64.2 ± 8.8					No significant decrease showed in SMM after NAC. Prevalence of sarcopenia: increased, from 47% to 53%.
								*Association with outcomes:*
								• The incidence of serious adverse events (e.g., febrile neutropenia) was associated with severe SML.
Guinan 2017 Ireland (42)	Prospective, To investigate SMM and physical performance from diagnosis to post-NAT.	EC	28 (23)	NAC: 21%	CT SMI (cm^2^/m^2^)	60.3 ± 8.1	54.7 ± 7.5	*Muscle change:*
		NS	62.8 ± 8.2	NCRT: 79%				SMM ↓ significantly after NAT. Prevalence of sarcopenia: increased, from 7% to 22%. *Association with outcomes*: NS
Motoori 2018 Japan (18)	Retrospective, To evaluate the influence of sarcopenia, changes in body composition, and adverse events during NCRT on postoperative infectious complications.	EC	83 (66)	NCRT: 100%	BIA SMI (kg/m^2^)	NS	NS	*Muscle change:*
		I-IV	65 (45-81)					SMM ↓ significantly after NCRT in patients with postoperative infectious complications. 53% patients had SML. Cut-off value for severe SML: >5%; 18.0% patients had severe SML.
								*Association with outcomes:*
								• SML during NCRT was a significant risk factor for postoperative infectious complications.
Jarvinen 2018 Finland (43)	Retrospective,	EC	115 (86)	NAC: 76%	CT SMI (cm^2^/m^2^)	NS	NS	*Muscle change:*
	To assess the effect of sarcopenia and skeletal muscle loss during NAT.	NS	NS	NCRT: 24%				Prevalence of sarcopenia: not significant increased, from 79% to 80%. Cut-off value for severe SML: >3%; 50% patients had severe SML.
								*Association with outcomes:*
								• Severe SML during NAT was associated with poor OS.
Ozawa 2019 Japan (29)	Retrospective, To investigate the impact of skeletal muscle loss on patients with ES after NAT.	EC	82 (71)	NAC: 46%	CT SM (PMI) (cm^2^/m^2^)	5.08 (2.74-9.93)^a^	4.87 (2.59-9.61)^a^	*Muscle change:*
		NS	63.5 ± 7.5	NCRT: 54%				Mean reduction in PMI value: 0.2 cm^2^/m^2^. 75.6% patients had SML.
								*Association with outcomes:*
								• Low muscle mass before surgery was related to higher risk of recurrence and poorer disease-free survival (DFS).
Yassaie 2019 New Zealand (30)	Retrospective, To assess whether the change in muscle mass with neoadjuvant treatment can predict postoperative outcomes.	EC	53 (49)	NAC: 89%	CT SM (TPA) (cm^2^)	NS	NS	*Muscle change:*
		0-IV	*SML ≤4%:* 62.6 ± 6.7	NCRT: 11%				Loss rate in TPA after NAT: 7.3 ± 6.8%. Cut-off value for severe SML: >4%; 62.3% had severe SML.
			*SML >4%:* 65.8 ± 8.0					
								*Association with outcomes:*
								• Severe SML was associated with higher risk of postoperative mortality.
Yoon 2020 Korea (37)	Retrospective, To assess whether sarcopenia and skeletal muscle loss affected survival outcomes of esophageal cancer patients who received NCRT followed by surgery.	EC	248 (NS)	NCRT: 100%	CT SMI (cm^2^/m^2^)	49.72 ± 7.92	45.10 ± 7.57	*Muscle change:*
		NS	63.5 ± 7.6					Change rate in SMM: -6.6 ± 6.1% Prevalence of sarcopenia: increased, from 63% to 84%. Cut-off value for severe SML: >10%; 28.2% patients had severe SML.
								*Association with outcomes*:
								• Severe SML was associated with poorer OS and DFS.
Kawakita 2020 Japan (26)	Retrospective, To investigate the effect of the severity and timing of changes in PMI on the survival of patients under NCRT plus esophagectomy and the association between PMI and other prognostic markers in these patients.	EC	113 (96)	NCRT: 100%	CT SM (PMI)	*SML*≥*20% after surgery (n=27):* 4.80 (3.38-5.81)^a^	*SML* ≥*20% after surgery (n=27):* 4.52 (3.16-5.03)^a^	*Muscle change:*
		IIb-IIIc	*SML <20%:* 64 (59-68)					Median (range) rate loss in PMI: 5.3 (1.5-12.7) % Cut-off value for severe SML: ≥13%; 25.0% patients had severe SML.
			*SML ≥20%:* 65 (56-68)		(cm^2^/m^2^)	*SML<20% after surgery (n=86):* 4.26 (3.72-5.41)^a^	*SML <20% after surgery (n=86):* 4.22 (3.55-4.95)^a^	*Association with outcomes*:
								• No significant association between muscle loss during NACT and OS or DFS was found
Hagens 2020 Netherlands (44)	Retrospective, To evaluate the change in body composition, sarcopenia, and muscle strength during NCRT, and the impact of body composition and muscle strength on postoperative morbidity and survival.	EC	322 (244)	NCRT: 100%	CT SMI (cm^2^/m^2^)	*No complication (n=86):* 46.2 ± 9.3	*No complication (n=94):* 46.3 ± 8.7	*Muscle change:*
		NS	63.7 ± 8.7					• Prevalence of sarcopenia: not significant increased, from 56% to 58%.
						*Minor complication (n=95):* 46.1 ± 8.6	*Minor complication (n=107):* 45.0 ± 8.5	*Association with outcomes*:
								• No significant association between muscle loss during NACT and postoperative morbidity was found.
						*major complication* (n=44): 46.9 ± 10.1	*major complication* (n=66): 46.3 ± 9.2	
Boer 2020 UK (27)	Retrospective, To assess changes in body composition during NAC and to determine its predictive value for postoperative complications.	EC	199 (158)	NAC: 100%	CT SMI (cm^2^/m^2^) SMA (m^2^)	51.87 ± 10.31	49.19 ± 9.71	*Muscle change:*
		AEGJ	66 (28-80)			150.41 ± 33.61	142.60 ± 31.94	SMM ↓ significantly after NAC. Prevalence of sarcopenia: increased, from 42% to 54%. Cut-off value for severe SML: >5%; 45.7% patients had severe SML.
		GC						
		NS						
								*Association with outcomes*:
								• No significant association between severe SML and postoperative complications was found.
Matsuura 2019 Japan (26)	Retrospective, To clarify whether low pre-treatment SMM could be a predictor of adverse events during NAC and explore the relationship between SMM and adverse events during NAC.	GC	41 (28)	NAC: 100%	CT SM (PMI) (cm^2^/m^2^)	4.77 ± 1.11	4.50 ± 1.20	*Muscle change:*
		II-IV	72 (48-82)					• SMM ↓ significantly after NAC: -5.95 ± 7.69%
								*Association with outcomes*:
								• Severe diarrhea was associated with SML during NAT.
Zhang 2021 China (25)	Retrospective, To explore the association between body composition changes during NAT and survival in patients with GC.	GC	157 (115)	NAC: 82%	CT SMA (cm^2^)	137.96 (111.87-154.41)^a^	137.97 (112.03-156.41)^a^	*Muscle change:*
		0-III	61 (53-67)	NCRT: 18%				No significant change showed in SMM after NAT. Cut-off value for severe SML: >2%; 42.7% patients had severe SML.
								*Association with outcomes*:
								• No significant association between skeletal muscle mass change and survival was found.
Levolger 2017 Netherlands (17)	Retrospective, To assess body composition changes during NCRT and its impact on outcome.	RC	122 (71)	NCRT: 100%	CT SMI (cm^2^/m^2^)	46.6 (41.2-53.4)^b^	46.9 (40.2-53.1)^b^	*Muscle change:*
		III, IV	61(53-66)					• No significant change showed in mean SMI after NCRT, while a wide distribution in muscle change was observed.
								*Association with outcomes*:
								• SML during NCRT was associated with DFS and distant metastasis-free survival.
Nardi 2019 Italy (23)	Retrospective, To establish the correlation between body composition changes after NCRT and postoperative outcomes.	RC	52 (34)	NCRT: 100%	CT SMA (cm^2^)	133.87 ± 31.6	133.39 ± 31.5	*Muscle change:*
		NS	63 (32-79)					No significant change showed in SMM after NCRT. Prevalence of sarcopenia: not significant increased, from 58% to 60%. 36.5% patients had SML >2%, and 30.7% >5%.
								*Association with outcomes*:
								• Severe SML during NCRT was associated with shorter DFS.
Fukuoka 2019 Japan (16)	Retrospective,	RC	47 (35)	NAC: 43%	CT SM (PMI) (cm^2^/m^2^)	325.4 (146.7-696.1)^c^	313.0 (110.5-722.3)^c^	*Muscle change:*
	To explore the relationship between skeletal muscle changes during NAT and prognosis.	I-III	66 (27-88)	NCRT: 57%				Mean change rate was -4.3%, and the range was -25.2-24.8%. Cut-off value for severe SML: >10%; 31.9% patients had severe SML.
								*Association with outcomes*:
								• Severe SML during NAT was associated with shorter DFS and OS.

^1^EC, esophageal cancer; GC, gastric cancer; CRC, colorectal cancer; AEGJ, adenocarcinoma of esophagogastric junction; NS, not specified.

^2^Age was presented as mean ± sd OR median (range).

^3^Treatment: NAC, neoadjuvant chemotherapy; NCRT, neoadjuvant chemoradiotherapy.

^4^FFM, fat free mass (calculated based on SMM); SMM, skeletal muscle mass; SMI, skeletal muscle index; SMA, skeletal muscle area; SML, skeletal muscle loss.

SM (PMI) = skeletal muscle which was evaluated as psoas muscle index; SM (TPA) = skeletal muscle which was evaluated as total psoas muscle area.

a Data was presented as median (range).

b Data was presented as median (IQR).

c Data was presented as mean (range).

The symbol ↓ means “muscle mass decreased after neoadjuvant therapy”.

**Table 3:** Main clinical outcomes included in meta-analysis (n = 8).

1st Author Year Country	Survival outcomes		Short-term outcomes (severe muscle loss *VS* No significant muscle loss)			
	OS	DFS	Total complications (yes/no)	Anastomotic leakage (yes/no)	Pneumonia (yes/no)	Mortality (yes/no)
Liu 2016 Japan (41)	2.78 (1.16-7.12)		20/34	6/48	6/48	1/53
			15/15	2/28	3/27	1/29
Jarvinen 2018 Finland (43)	1.64 (1.00-3.37)		62/30	13/79		9/83
			17/6	2/21		1/22
Ozawa 2019 Japan (29)		1.00 (0.40-2.16)	4/14			
			19/45			
Yassaie 2019 New Zealand (30)				6/27	15/18	8/25
				3/17	7/13	0/20
Yoon 2020 Korea (37)	2.23 (1.42-3.73)					
Boer 2020 UK (27)			45/46	1/90	23/68	2/89
			56/52	7/101	29/79	3/105
Levolger 2017 Netherlands (17)		1.04 (1.01-1.06)				
Fukuoka 2019 Japan (16)		5.78 (1.68-19.93)	11/4			
			15/17			

OS overall survival, DFS disease-free survival were presented as Hazard Radio (95% Confidence Interval).

### Quality Assessment

Eight studies were of good quality (16, 18, 25–27, 36, 37, 41), and the others (17, 23, 24, 29, 30, 38–40, 42–44) were regarded as fair. However, it should be noted that all studies did not provide a sample size justification. The quality assessments of the included studies are summarized in **Table 4**.

**Table 4:** Quality assessment for included studies based on NIH Quality Assessment Tool for Observational Cohort and Cross-Sectional Studies (n = 19).

		Mayanagi 2017	Ida 2014 (38)	Reisinger 2015 (40)	Liu 2016 (41)	Miyata 2017 (24)	Guinan 2017 (42)	Motoori 2018 (18)	Jarvinen 2018 (43)	Ozawa 2019 (29)	Yassaie 2019 (30)	Yoon 2020 (37)	Kawakita 2020 (26)	Hagens 2020 (44)	Boer 2020 (27)	Matsuura 2019 (36)	Zhang 2021 (25)	Levolger 2017 (17)	Nardi 2019 (23)	Fukuoka 2019 (16)
1	Was the research question or objective in this paper clearly stated?	Y	Y	Y	Y	Y	Y	Y	Y	Y	Y	Y	Y	Y	Y	Y	Y	Y	Y	Y
2	Was the study population clearly specified and defined?	Y	Y	Y	Y	Y	Y	Y	Y	Y	Y	Y	Y	Y	Y	Y	Y	Y	Y	Y
3	Was the participation rate of eligible persons at least 50%?	Y	Y	Y	Y	N	Y	Y	Y	Y	N	Y	Y	Y	NR	Y	Y	N	NR	Y
4	Were all the subjects selected or recruited from the same or similar populations?	Y	Y	Y	Y	Y	Y	Y	Y	Y	Y	Y	Y	Y	Y	Y	Y	Y	Y	Y
5	Was a sample size justification, power description, or variance and effect estimates provided?	N	N	N	N	N	N	N	N	N	N	N	N	N	N	N	N	N	N	N
6	For the analyses in this paper, were the exposure(s) of interest measured prior to the outcome(s) being measured?	Y	Y	Y	Y	Y	Y	Y	Y	Y	Y	Y	Y	Y	Y	Y	Y	Y	Y	Y
7	Was the timeframe sufficient so that one could reasonably expect to see an association between exposure and outcome if it existed?	Y	Y	Y	Y	Y	NR	Y	Y	Y	Y	Y	Y	Y	Y	Y	Y	Y	Y	Y
8	For exposures that can vary in amount or level, did the study examine different levels of the exposure as related to the outcome (e.g., categories of exposure, or exposure measured as continuous variable)?	N	N	N	Y	N	N	Y	Y	N	Y	Y	Y	N	Y	Y	Y	N	Y	Y
9	Were the exposure measures (independent variables) clearly defined, valid, reliable, and implemented consistently across all study participants?	Y	Y	Y	Y	Y	Y	Y	Y	Y	Y	Y	Y	Y	Y	Y	Y	Y	Y	Y
10	Was the exposure(s) assessed more than once over time?	Y	Y	Y	Y	Y	Y	Y	Y	Y	Y	Y	Y	Y	Y	Y	Y	Y	Y	Y
11	Were the outcome measures (dependent variables) clearly defined, valid, reliable, and implemented consistently across all study participants?	Y	Y	Y	Y	Y	Y	Y	Y	Y	Y	Y	Y	Y	Y	Y	Y	Y	Y	Y
12	Were the outcome assessors blinded to the exposure status of participants?	NA	NA	NA	NA	NA	NA	NA	NA	NA	NA	NA	NA	NA	NA	NA	NA	NA	NA	NA
13	Was loss to follow-up after baseline 20% or less?	Y	Y	Y	Y	Y	Y	Y	Y	Y	Y	Y	Y	Y	Y	Y	Y	Y	Y	Y
14	Were key potential confounding variables measured and adjusted statistically for their impact on the relationship between exposure(s) and outcome(s)?	Y	N	N	Y	N	N	Y	Y	Y	N	Y	Y	Y	Y	Y	Y	Y	N	Y
**Conclusion (A: Good; B: Fair; C: Poor)**		B	B	B	A	B	B	A	B	B	B	A	A	B	A	A	A	B	B	A

Y, yes; N, no; NR, not reported; NA, not applicable.

### Effect of Neoadjuvant Therapy on Skeletal Muscle Mass

The skeletal muscle indicators involved in the review include skeletal muscle mass (SMM), skeletal muscle index (SMI), skeletal muscle area (SMA), psoas muscle index (PMI), and total psoas muscle area (TPA), and only the study of Yip et al. (39) used fat free mass (FFM) to represent skeletal muscle. In this study, FFM was estimated using a regression equation based on skeletal muscle mass, and thus we included it in our study. In addition, some studies determined the specific cut-off value for the rate of muscle loss to divide patients into severe loss group and no significant muscle loss group. However, the value varied from 3% to 13% due to different calculation methods. Two studies (18, 43) set the value based on previous studies, and four (16, 25, 30, 37) used the ROC curve, with the remaining three studies (26, 27, 41) used specific percentile values to determine the cut-off value.

**Figure 2:**
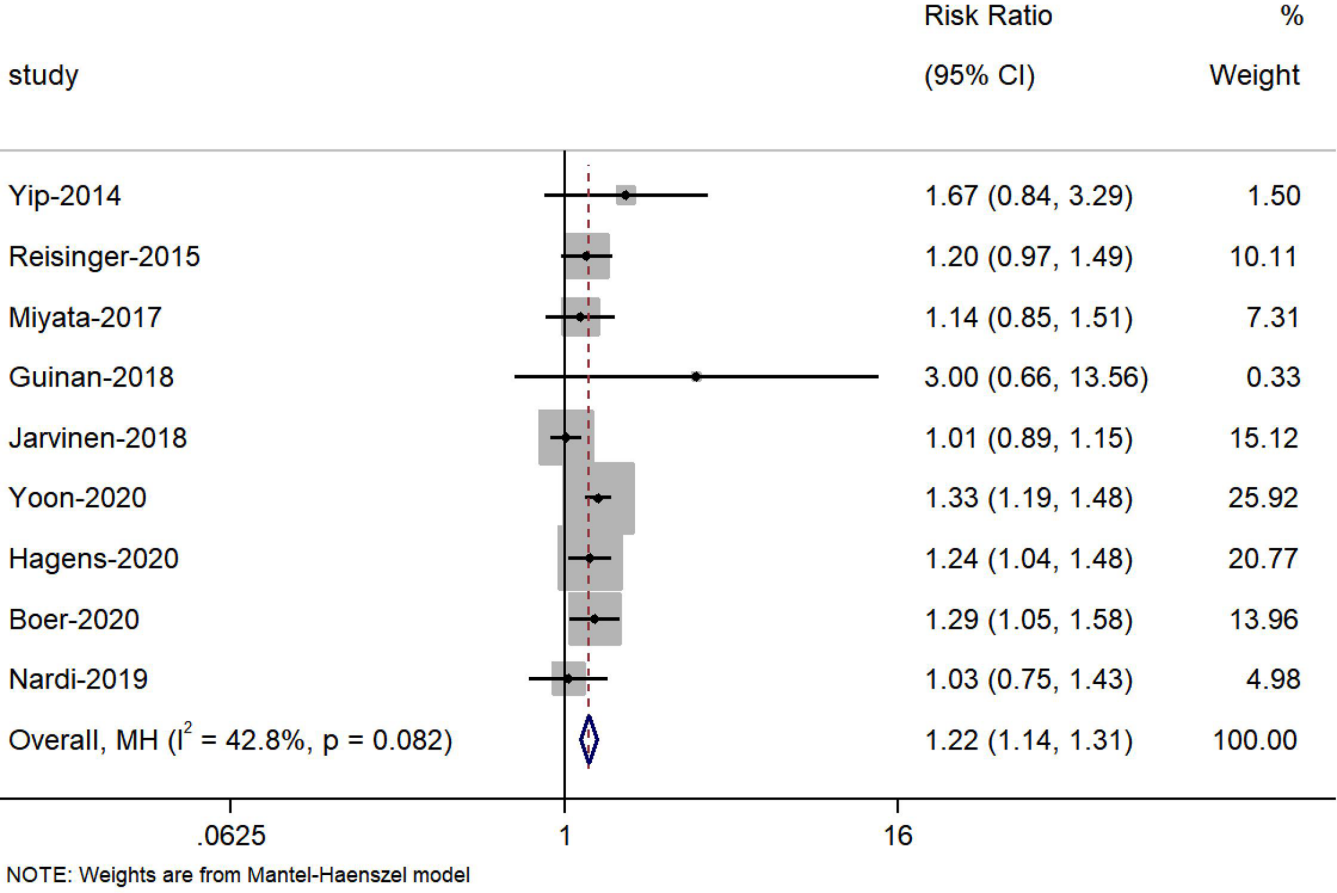
Forest plots of the prevalence of sarcopenia before and after neoadjuvant therapy.

**Figure 3:**
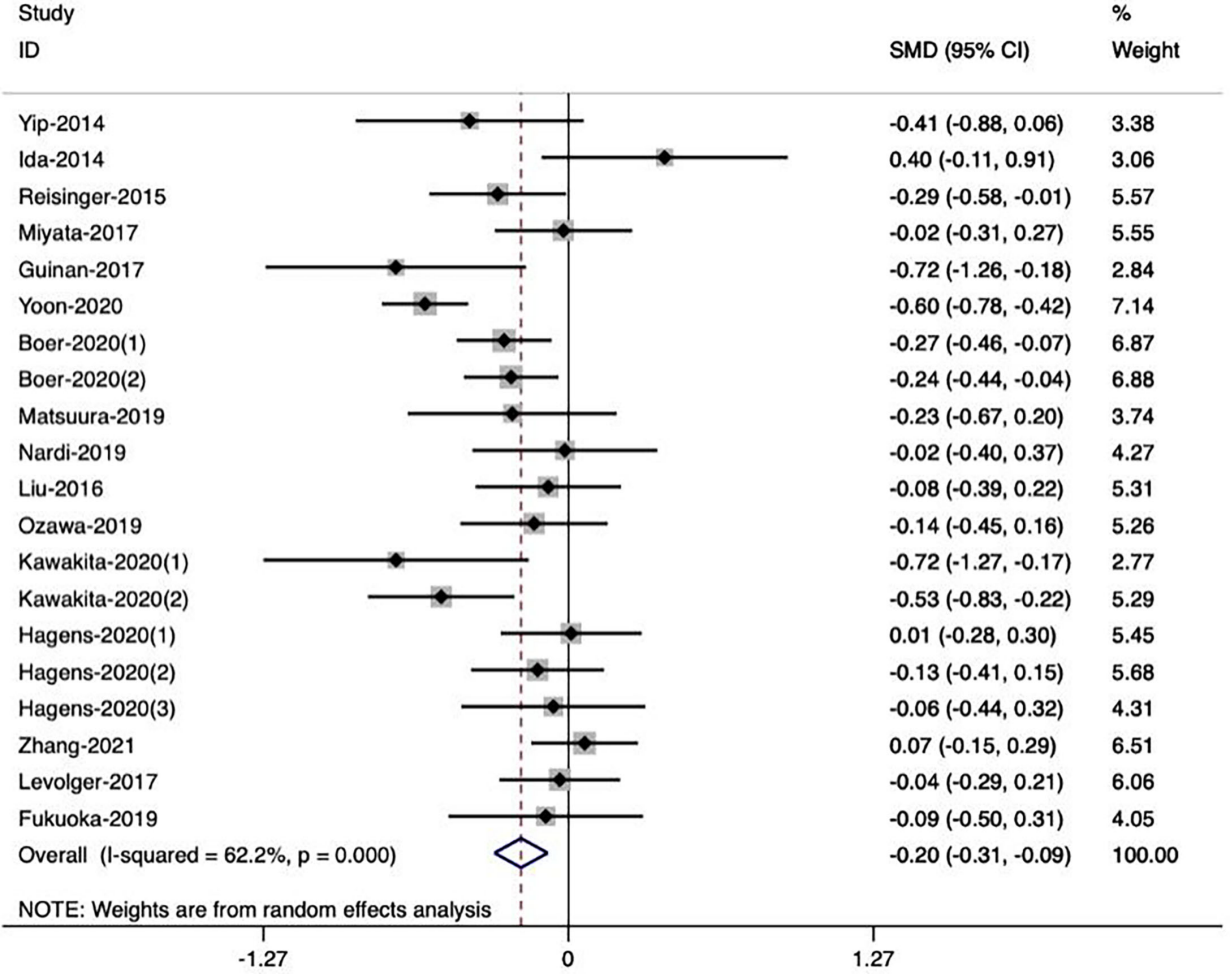
Forest plots of the effect of neoadjuvant therapy on skeletal muscle mass

Nine studies (23, 24, 27, 37, 39, 40, 42–44) reported the change in the prevalence of sarcopenia during NAT, and the meta-analysis showed that the risk of sarcopenia increased 22% after receiving NAT (HR=1.22, 95% CI 1.14, 1.31, Z=4.286, P<0.001) **(** **Figure 2** **)**. Meanwhile, seven studies (18, 27, 36, 39–42) reported a significant change of skeletal muscle during NAT, while the other twelve studies observed the opposite result (16, 17, 23–26, 29, 30, 37, 38, 43, 44). Bases on the extracted data, we included sixteen studies (23, 24, 27, 36–40, 42) with 20 groups of sufficient data in our meta-analysis to explore the effect of NAT on skeletal muscle. In the random effects model, NAT was associated with skeletal muscle loss, with a standardized mean difference of -0.20 (95% CI -0.31, -0.09, *Z*=3.49, P<0.001). However, there was a moderate heterogeneity between the studies (*I^2^ = *62.2%, *P*<0.001) **(** **Figure 3** **)**. As such, we conducted multiple meta regression according to the study design, population, cancer type, neoadjuvant therapy type, and measuring tool. The results indicate that population, neoadjuvant therapy type, and measuring tool were the potential sources of heterogeneity (**Table 5**). From the following subgroup analysis results **(** **Supplement Figure S1** **)**, skeletal muscle mass decreased significantly in the non-Asian population group, NCRT group and CT group (random effect models: SMD = -0.19, 95% *CI* -0.28, -0.09, *Z*=3.69, P<0.001; SMD = -0.25, 95% *CI* -0.44, -0.07, *Z*=2.66, P=0.008; SMD = -0.23, 95% *CI* -0.34, -0.12, *Z*=3.99, P<0.001, respectively).

**Table 5:** Results of multiple meta regression for potential source of heterogeneity.

Variable	Regression coefficient (SE)	95%*CI*	*t*	*P* value
**Study design**				
retrospective *VS* prospective	-0.19 (0.16)	(-0.54, 0.15)	-1.23	0.246
**Population**				
Asian *VS* Non-Asian	0.38 (0.12)	(0.12, 0.65)	3.21	**0.008**
**Cancer type**				
esophageal *VS* gastric	-0.33 (0.16)	(-0.67, 0.01)	-2.14	0.055
gastro- esophagus *VS* gastric	-0.30 (0.23)	(-0.80, 0.20)	-1.31	0.216
rectum *VS* gastric	-0.21 (0.20)	(-0.64, 0.22)	-1.10	0.296
**NAT type**				
NAC *VS* NAC+NCRT	-0.43 (0.21)	(-0.90, 0.04)	-2.02	0.069
NCRT *VS* NAC+NCRT	-0.29 (0.13)	(-0.56, -0.01)	-2.29	**0.043**
**Measuring. tool**				
CT *VS* BIA	0.80 (0.26)	(0.21, 1.38)	3.01	**0.012**

Bold values mean here is significant difference between the two variables (P<0.05).

### Relationship Between Muscle Loss During Neoadjuvant Therapy and Postoperative Outcomes

#### Long-Term Survival Outcomes

Nine studies (16, 17, 23, 26, 37, 39, 41, 43, 44) reported the relationship between muscle loss during NAT and postoperative overall survival. Five studies (16, 37, 41, 43, 44) showed that the group with muscle loss had a poorer overall survival than the group without, while the others did not find a significant difference between them. For disease-free survival, five studies (16, 18, 23, 29, 37) demonstrated that it was associated with the decrease in muscle mass during NAT, but two studies (26, 40) could not observe the difference.

**Figure 4:**
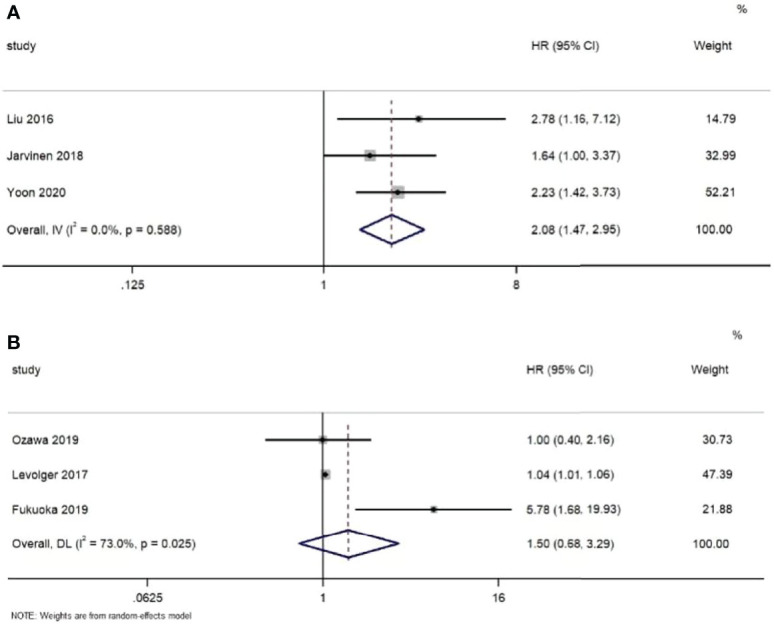
Forest plots of the relationship between muscle loss during neoadjuvant therapy and **(A)** overall survival and **(B)** disease-free survival

Meanwhile, six studies (16, 17, 29, 37, 41, 43) provided sufficient data on survival outcomes (**Table 3**). Based on the extracted data, we conducted the meta-analyses. As shown in **Figure 4**, the results indicate that in the fixed effects model, muscle loss during NAT was significantly related to overall survival (HR=2,08, 95% CI =1.47, 2.95, *Z*=4.12, P<0.001, *I^2 ^= *0.0%). However, in the random effects model, no significant association was found with disease-free survival (HR=1.50, 95% CI =0.68, 3.29, *Z*=1.00, P=0.317, *I^2 ^= *73.0%).

#### Postoperative Complications

There were seven studies (16, 25, 27, 29, 38, 41, 43) concerning the effect of muscle loss during NAT on postoperative total complications, while only one study (38) demonstrated that total complications were associated with marked muscle loss and the others showed no significance. Two studies (16, 27) involved severe complications, which was defined as Clavien-Dindo graded 3 (45), though the results were not consistent. Except for total complications and severe complications, some studies also demonstrated specific complications, including blood loss (41), anastomotic leakage (27, 30, 41), pneumonia (27, 29, 30, 41), recurrent nerve paralysis (41) and chylothorax (30, 41), but none of the results had a significant difference. However, when considering postoperative infection, there were two studies showing that muscle loss during NAT was associated with a higher rate of infection (18, 27).

**Figure 5:**
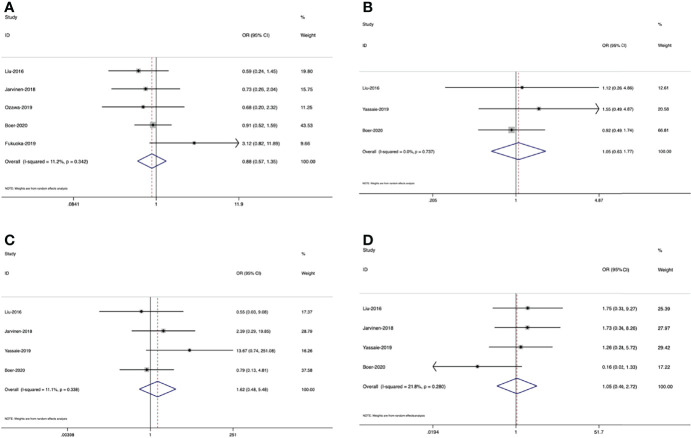
Forest plots of the relationship between muscle loss during neoadjuvant therapy and **(A)** total complications, **(B)** anastomotic leakage, **(C)** pneumonia and **(D)** mortality

There were five studies (16, 27, 29, 41, 43), 3 (27, 30, 41) and 4 studies (27, 30, 41, 43) separately included in the meta-analyses of the relationship between muscle loss and total complications, pneumonia, and anastomotic leakage (**Table 3**). The pooled results in the random effects model revealed that there was no significant association between muscle loss and these postoperative outcomes. (**Figure 5B** **(**OR=0.88, 95% CI =0.57, 1.35, *Z*=0.6, P=0.55), **Figure 5C** (OR=1.05, 95% CI =0.63, 1.77, *Z*=0.19, P=0.85), **Figure 5A** (OR=1.62, 95% CI =0.48, 5.48, *Z*=0.77, P=0.44)

#### Postoperative 30-d Mortality

There were four studies (27, 30, 40, 41) illustrating the association between loss of muscle mass and postoperative 30-d mortality. One study (30) supported that decreased muscle mass during neoadjuvant therapy could be a predictor of increased mortality in patients receiving esophageal cancer resection, while two studies (27, 41) could not support this conclusion. Another study (40) found that in patients with stage III–IV tumors, there was a significant difference between 30-d mortality and muscle loss, but this difference did not exist in patients with stage I-II tumors. However, the result of meta-analysis demonstrated that muscle loss was not related to postoperative mortality. [**Figure 5D** **(**OR=1.05, 95% CI =0.40, 2.72, *Z*=0.1, P=0.922]

#### Length of Hospital Stay

Three studies (29, 40, 41) mentioned length of hospital stay and these results all demonstrated that there were no significant differences between the muscle loss and non-muscle loss groups.

### Publication Bias and Sensitivity Analysis

**Figure 6:**
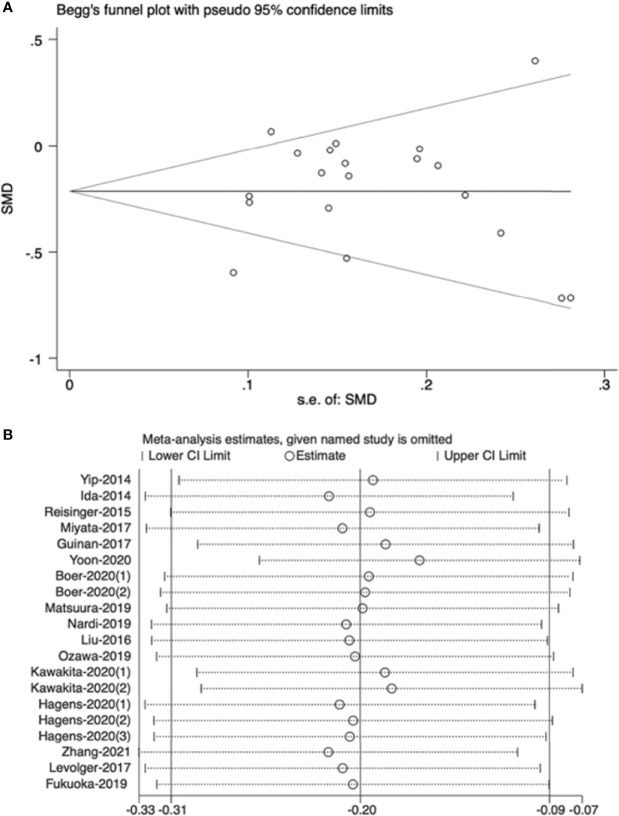
**(A)** Publication bias of effect of neoadjuvant therapy on muscle mass, **(B)** Sensitivity analysis of effect of neoadjuvant therapy on muscle mass

The funnel plot revealed that there was no high publication bias in these studies (Begg’s test, P=0.544). Furthermore, we conducted a sensitivity analysis for the meta-analysis of effect of NAT on muscle mass and the results were stable when separately excluding studies. The results are shown in **Figure 6**.

## Discussion

This systematic review and meta-analysis was the first to investigate the skeletal muscle change during neoadjuvant therapy in patients with gastrointestinal cancers. The pooled results suggest that NAT significantly reduced skeletal muscle mass and muscle loss during NAT could be a prognostic indicator for overall survival.

Body weight (BW) and body mass index (BMI) are the most widely used indicators to evaluate nutrition status, which can be easily assessed in clinical practice (46). However, due to the high prevalence of chemotherapy-induced oedema (47), BW, or BMI may not be the reliable and sensitive indicator for patients receiving NAT. It is notable that most studies included in our study only found the decrease of skeletal muscle mass after NAT without any significant change in BW or BMI. Patients with GI cancers were reported to maintain or even gain body weight during NAT (36, 42), while more than 50% patients experienced skeletal muscle loss (18, 29, 41), and the mean rate of loss was around 5% (16, 26, 39). Our meta-analysis also indicated that NAT was significantly correlated with skeletal muscle loss and the results were proved to be stable without any publication bias. Thus, more attention should be paid to the change of skeletal muscle during NAT. Currently, the detailed mechanisms for skeletal muscle loss during NAT remain uncertain but chemotherapy-related toxicities can be one related reason (24). Toxicities not only cause direct acute damage to mitochondrial function, but also increase the risk of intestinal inflammation including diarrhea, nausea, and vomiting (48–51). As the increasing energy consumption caused by intestinal inflammation, skeletal muscle protein catabolism is enhanced (52). Matsuura et al. (36) found that patients who experienced severe diarrhea showed more skeletal muscle loss. Therefore, prevention of those symptoms may help to preserve muscle mass during NAT. Additionally, the relationship between muscle loss and the effectiveness of NAT should be explored in future study.

In our study, the population was one of the potential sources of heterogeneity based on meta regression. The Asian population has a lower BMI and lower prevalence of sarcopenia than non-Asian individuals (52). As such, the degree of muscle change can vary significantly by population. Moreover, NAT dominates the treatment strategies for GI cancers in western countries, while surgery is still the main method in Asia (53). There are some differences in the neoadjuvant treatment protocols (40, 41). Therefore, future studies need to explore the pattern of muscle change during NAT according to different populations. Meanwhile, the type of NAT was considered as a source of heterogeneity. NAT mainly includes neoadjuvant chemotherapy (NCT), neoadjuvant chemoradiotherapy (NCRT), and NCT combined with NCRT. Although there were no significant differences among these three therapies on improving short-term and long-term outcomes in patients with GI cancers (54, 55), our findings indicate that they had inconsistent impact on skeletal muscle. As such, researchers can further compare different types based on their effect on skeletal muscle and provide evidence for the best choice of treatment regimens. In addition, our results show that measuring tools could cause heterogeneity. Both CT and BIA are effective tools to assess body composition recommended by guidelines (56, 57), but some studies argued the accuracy of skeletal muscle mass assessment by BIA, especially for patients with severe ascites or oedema (58). However, CT requires expensive equipment, has high amount of radiation exposure, and the analysis of body composition from CT necessitates specialist software and training, while BIA is practical, non-invasive, and convenient to perform (59). Therefore, future studies should identify the most suitable tools when assessing muscle change for patients with GI cancers. The assessment of muscle mass should also be included into the regular standard of care for patients who are receiving NAT. We also found that different cancer types were not a potential source of heterogeneity in this study, while previous studies reported that patients with upper-GI cancer, especially esophageal cancer, were at a higher risk of malnutrition (60). The possible reason may be associated with the tumor obstruction in the esophagus (61), leading to the high prevalence of dysphagia and weight loss. Considering this significant syndrome and its negative impact on nutrition condition, researchers could compare the degree of muscle loss during NAT based on different types of GI cancer in the future.

Several systematic reviews have summarized the predictive value of preoperative sarcopenia or sarcopenia at diagnosis in patients with GI cancers (10, 22, 62). However, many included studies reported that the degree of sarcopenia was aggravated and a significant number of patients became sarcopenic during NAT, indicating the continuous change state of body composition and nutrition condition (24, 37, 40, 42). Hence, it is more meaningful to focus on the effect of skeletal muscle changes during NAT rather than pre-NAT or post-NAT muscle mass. We conducted qualitative and quantitative synthesis to explore the impact of muscle loss on both long-term and short-term clinical outcomes. On the aspect of long-term survival, the meta-analysis results showed a significant and strong association between muscle loss during NAT and overall survival, suggesting that skeletal muscle loss during neoadjuvant therapy could be a prognostic indicator for overall survival. However, no correlation with disease-free survival was found. The possible reasons may be related to the limited amount of included articles and high heterogeneity. Conversely, we demonstrated that severe muscle loss during NAT was not associated with short-term postoperative outcomes, including length of hospital stay, postoperative complications, and 30-d morbidity. It is notable that the following curative surgery resection may also reduce skeletal muscle mass and several studies have reported that severe acute surgery-induced muscle loss is related to longer a hospital stay, more postoperative complications, and poorer quality of life (63, 64). As such, worse short-term postoperative outcomes may be attributed to postoperative muscle loss rather than loss during NAT, and more related studies are needed to validate this conclusion. Currently, there is no gold standard to determine severe muscle loss during NAT and the cut-off value varies in our included studies. The identification of cut-off value is one direction of further study, which is beneficial to screen the risk factors for severe muscle loss and implement early detection and intervention.

Due to the close relationship between muscle loss during NAT and worse survival, target intervention should be conducted to prevent severe muscle loss during NAT. As we referred to in the “Introduction”, there are multifactorial causes of muscle loss for patients with GI cancers and patients usually suffer from lower energy intake and higher energy expenditure (65). Symptoms like sarcopenia, dysphagia, malnutrition, and osteoporosis can overlap in the individuals, especially in elderly or frail patients (66). Therefore, comprehensive and multidisciplinary management is crucial in clinical practice and alternative strategies for muscle loss in cancer patients mainly include exercise training, nutritional supplementation, appetite stimulants, and other developing pharmacological agents (67, 68). Among them, exercise training is recommended as an effective non-pharmacological intervention to reduce muscle loss and improve muscle function. One systematic review (69) demonstrated that resistance training could improve mitochondrial density and dynamics, while endurance training was related to increased mitochondrial antioxidant capacity, which can provide strong evidence for clinical target exercise intervention. Currently, there are several drugs being tested in the preclinical or at Phase III stage, which have great possibility to preserve muscle and fat mass and prolong survival (68). As such, a balanced combination of pharmacological and non-pharmacological interventions should be an important goal in the future.

Some limitations should be acknowledged in this study. Firstly, all included studies were observational study in English, and more than half of studies were graded as moderate quality, which may affect the strength of evidence and increase the risk of bias. Secondly, several studies performed Kaplan-Meier method and log-rank test to analyze the effect of muscle loss on clinical outcomes. Therefore, we failed to obtain sufficient data of HRs and ORs for those outcomes. In addition, when the association between muscle loss and outcomes was summarized, only a small number of articles were included in our meta-analysis and subgroup analysis were not stratified.

## Conclusion

In summary, this systematic review and meta-analysis revealed that skeletal muscle decreased significantly during neoadjuvant therapy in patients with gastrointestinal cancers and skeletal muscle loss was strongly associated with worse overall survival. Future research should identify the criteria of severe skeletal muscle and further explore the effect on short-term clinical outcomes. Moreover, high-quality prospective studies and randomized controlled trials based on specific population, neoadjuvant treatment, measuring tool and cancer type are needed.

## Data Availability Statement

The original contributions presented in the study are included in the article/**Supplementary Material**. Further inquiries can be directed to the corresponding author.

## Author Contributions

QX contributed to study design. X-YX and X-MJ were responsible for literature search, data extraction and analysis and wrote the manuscript. S-QZ contributed to study selection. HX, J-HL, CY, and QX revised and polished the article. All authors contributed to the article and approved the submitted version.

## Funding

This study was supported by Project “The exploration of trajectories and intervention program of frailty for gastric cancer survivors based on the health ecology theory” supported by National Natural Science Foundation of China (NSFC) (No.82073407).

## Conflict of Interest

The authors declare that the research was conducted in the absence of any commercial or financial relationships that could be construed as a potential conflict of interest.

## Publisher’s Note

All claims expressed in this article are solely those of the authors and do not necessarily represent those of their affiliated organizations, or those of the publisher, the editors and the reviewers. Any product that may be evaluated in this article, or claim that may be made by its manufacturer, is not guaranteed or endorsed by the publisher.

## Supplementary Material

Supplementary Figure 1 Subgroup analysis of muscle change based on **(A)** population; **(B)** NAT type; **(C)** measuring tool.
