# Supplementary figures and images for: Skeletal Muscle Change During Neoadjuvant Therapy and Its Impact on Prognosis in Patients With Gastrointestinal Cancers: A Systematic Review and Meta-Analysis
Source: Front Oncol. 2022 May 27;12:892935. doi: 10.3389/fonc.2022.892935 (PMC9186070; doi:10.3389/fonc.2022.892935)

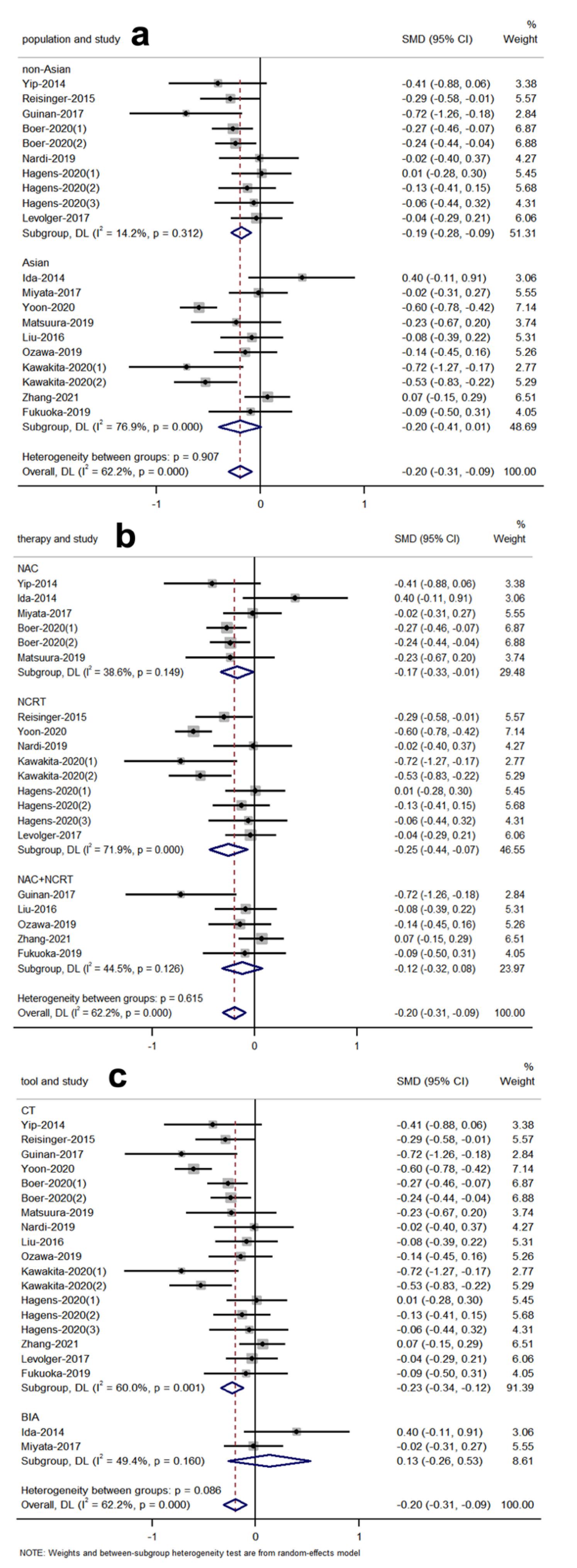

Supplement: Supplementary Figure 1 — Subgroup analysis of muscle change based on (A) population; (B) NAT type; (C) measuring tool. [file Image_1.jpeg]
